# Does low-temperature dialysis still represent an advantage?: a narrative review from the last decade

**DOI:** 10.1007/s11255-025-04850-2

**Published:** 2025-10-21

**Authors:** Lorenzo D’Elia, Deborah Di Vico, Giovanni Otranto, Annalisa Villani, Luca Di Lullo, Antonio Bellasi, Vincenzo Barbera

**Affiliations:** 1 Department of Nephrology and Dialysis, Ospedale L. Parodi Delfino, Azienda USL Roma 5, Colleferro, Italy; 2 Department of Nephrology and Dialysis, Azienda USL Roma 6, Albano Laziale, Italy; 3 Service of Nephrology, Ospedale Regionale di Lugano, Ospedale Civico, Ente Ospedaliero Cantonale (EOC), Via Tesserete 46, CH-6903 Lugano, Switzerland; 4 Faculty of Biomedical Sciences, Università della Svizzera italiana (USI), 6900 Lugano, Switzerland

**Keywords:** Cool dialysis, Low-temperature dialysate, Hemodynamic stability, Intradialytic hypotension, Cardiac protection

## Abstract

**Background:**

Low-temperature hemodialysis (*cool dialysis*) is a therapeutic strategy aimed at preventing intradialytic hypotension and improving hemodynamic stability in patients with end-stage kidney disease (ESKD). Over the past decade, several studies have investigated its potential impact on cardiovascular outcomes, neurological protection, and clinical tolerability, with sometimes conflicting results.

**Methods:**

An overview of the literature was conducted using PubMed® from January 1, 2015 to July 31, 2025, with the search terms *cooler dialysis*, *cooler dialysate*, and *cooler dialysate and hemodynamic stability*. Randomized and prospective clinical studies assessing the effects of low-temperature hemodialysis on hemodynamic, cardiac, and neurological outcomes were included.

**Results:**

Nine studies met the inclusion criteria. Small-scale trials reported a significant reduction in intradialytic hypotension episodes and a protective effect on left ventricular function. The large multicenter MyTEMP trial, while showing no significant reduction in major cardiovascular events, confirmed a trend toward improved blood pressure stability, at the expense of increased thermal discomfort. A Bayesian reanalysis of MyTEMP data suggested a high probability of clinical benefit. Effects on cerebral microcirculation and cognitive function remain uncertain.

**Conclusions:**

Low-temperature hemodialysis is associated with improved hemodynamic stability and potential myocardial protection in specific patient subgroups; however, consistent effects on major cardiovascular events or cognitive decline in the general population have not been observed. Large, multicenter studies with extended follow-up are needed to better define clinical indications, long-term outcomes, and the identification of “responder” patients.

## Introduction

Hemodialysis is a life-saving therapy for patients with end-stage kidney disease (ESKD). Nevertheless, 20–40% of patients die within the first year of initiating renal replacement therapy, mainly from cardiovascular causes [1–3]. Several imaging studies have demonstrated that hemodialysis can promote subclinical injury to the heart, brain, and other vital organs, primarily through recurrent episodes of intradialytic hypotension and consequent regional ischemia [4, 5].

Among the mechanisms involved, hemodynamic fluctuations that occur during each dialysis session play a key role. A drop in arterial blood pressure of up to 20 mmHg may lead to coronary hypoperfusion and impaired myocardial contractility, contributing to the phenomenon of myocardial stunning. This condition, characterized by a delayed recovery of regional myocardial function after ischemia–reperfusion, may occur even in the absence of irreversible myocardial damage [6–8].

The pathogenesis of these events is multifactorial and includes depletion of high-energy phosphates, microvascular dysfunction, dysregulation of calcium homeostasis, neurosensory alterations, and increased production of reactive oxygen species (ROS) [9]. Beyond the heart, hemodialysis-related hypoperfusion, oxidative stress, and systemic inflammation can also impair cerebral perfusion and contribute to neurological injury.

Given this background, strategies aimed at improving intradialytic hemodynamic stability have become a major clinical priority. Among them, low-temperature dialysis—or cool dialysis—has been proposed as a promising approach to attenuate vasodilation, reduce hypotensive episodes, and limit tissue hypoperfusion with its long-term consequences.

## Methods

A narrative review of the literature was conducted to evaluate the clinical impact of low-temperature hemodialysis (cool dialysis) on hemodynamic stability, cardiovascular outcomes, and neurological function. The small number of published studies relevant to this topic and their methodological heterogeneity preclude meaningful synthesis as a formal systematic review or meta-analysis at this stage. Therefore, we conducted a targeted literature search, focusing on one medical repository (PubMed database), to identify the most relevant and accessible evidence. This approach aligns with the scope and goals of our manuscript, which aimed to provide a concise synthesis and critical assessment of the available evidence rather than to create a comprehensive systematic review. We acknowledge the limitation that restricting the search to a single repository may overlook some studies indexed elsewhere, and we have noted this limitation in the manuscript. A formal, protocol-driven systematic review covering multiple databases could be undertaken as a separate future project if the literature base expands.

The PubMed® database was searched for articles published between January 1, 2015 and July 31, 2025 using the following keywords: *cooler dialysis*, *cooler dialysate*, and *cooler dialysate and hemodynamic stability*. Only studies conducted in adult populations and published in English were considered for this narrative review (Table 1).

**Table 1:** Summary of clinical studies on low-temperature hemodialysis (2015–2025)

Author / Year	Study design	N (patients)	Dialysate temperature protocol	Main outcomes	Key findings
Veerappan et al., 2015	Prospective crossover	60	36.5 ± 0.2 °C (range 35.7–37.5), personalized	Intradialytic hypotension (IDH), BP variability	↓ IDH episodes, improved hemodynamic stability
MyTEMP Trial (2017–2021)	Pragmatic, cluster-RCT, multicenter (Ontario)	> 15,000 (84 centers, > 4.3 M sessions)	Personalized (0.5–0.9 °C below baseline core temp, min 35.5 °C) vs. standard 36.5 °C	CV death, MI, stroke, CHF hospitalization	No significant reduction in major CV events; trend to improved BP stability; ↑ thermal discomfort
Ouyang et al., 2022 (Bayesian reanalysis of MyTEMP)	Post hoc Bayesian analysis	> 15,500	Same as MyTEMP	Composite CV endpoint	High probability of clinical benefit despite non-significant frequentist results
Odudu et al., 2020	Randomized controlled trial	73	Personalized dialysate temp vs. standard	LV systolic function, IDH	↓ IDH, attenuated transient LV dysfunction, cardioprotective effect
Dasgupta et al., 2023	Randomized controlled pilot study	~ 100 (pilot)	Cool dialysis vs. standard	Cognitive performance (MoCA, WAIS), tolerance	No significant cognitive differences at 12 months; intervention well tolerated
Eldehni et al., 2015	Imaging study	70 (approx.)	Cool dialysis (35–36 °C) vs. standard	Brain microcirculation (DTI-MRI)	Less microvascular white matter damage with cool dialysis
Yu et al., 2019	Cross-sectional neuroimaging	120	Standard vs. cool dialysis (subgroup)	Cortical thickness/volume (MRI)	ESRD patients show reduced cortical thickness; possible attenuation with cool dialysis
Sedaghat et al., 2016 (Rotterdam Study)	Population-based cohort with dialysis subgroup	> 500 (dialysis subgroup)	Not standardized	Cerebral autoregulation	Impaired autoregulation in dialysis; cooled dialysate may mitigate
FHN Trial, 2012 (exploratory link)	RCT, not designed for temp but relevant	245	Standard vs. intensive dialysis (temp not primary variable)	LV mass, cardiac outcomes	Suggested role of greater hemodynamic stability (indirect support for cooled dialysate)

We searched for randomized controlled trials and prospective observational studies that specifically evaluated the effects of reduced-temperature dialysate (defined as ≥ 0.5 °C below the standard 37 °C or tailored to the patient’s baseline core temperature) on:

1. Intradialytic blood pressure stability and occurrence of intradialytic hypotension (IDH);
2. Myocardial function and cardiovascular outcomes;
3. Cerebral microcirculation, cognitive function, and neurological endpoints.

Studies were excluded if they: (a) focused exclusively on pediatric patients, (b) did not report clinical outcomes related to hemodynamic or cardiovascular/neurological function, or (c) were narrative reviews, case reports, or editorials. Pediatric studies were not included in the final analysis but may be cited in the Introduction or Discussion for background purposes.

The initial search retrieved 14 articles, but 5 were excluded for not meeting the inclusion/exclusion criteria (e.g., reporting surrogate outcomes not aligned with the objectives of this review). A total of 9 studies were examined, summarized, and discussed in this narrative review.

### Impact of dialysis on the cardiovascular system

**Fig. 1:**
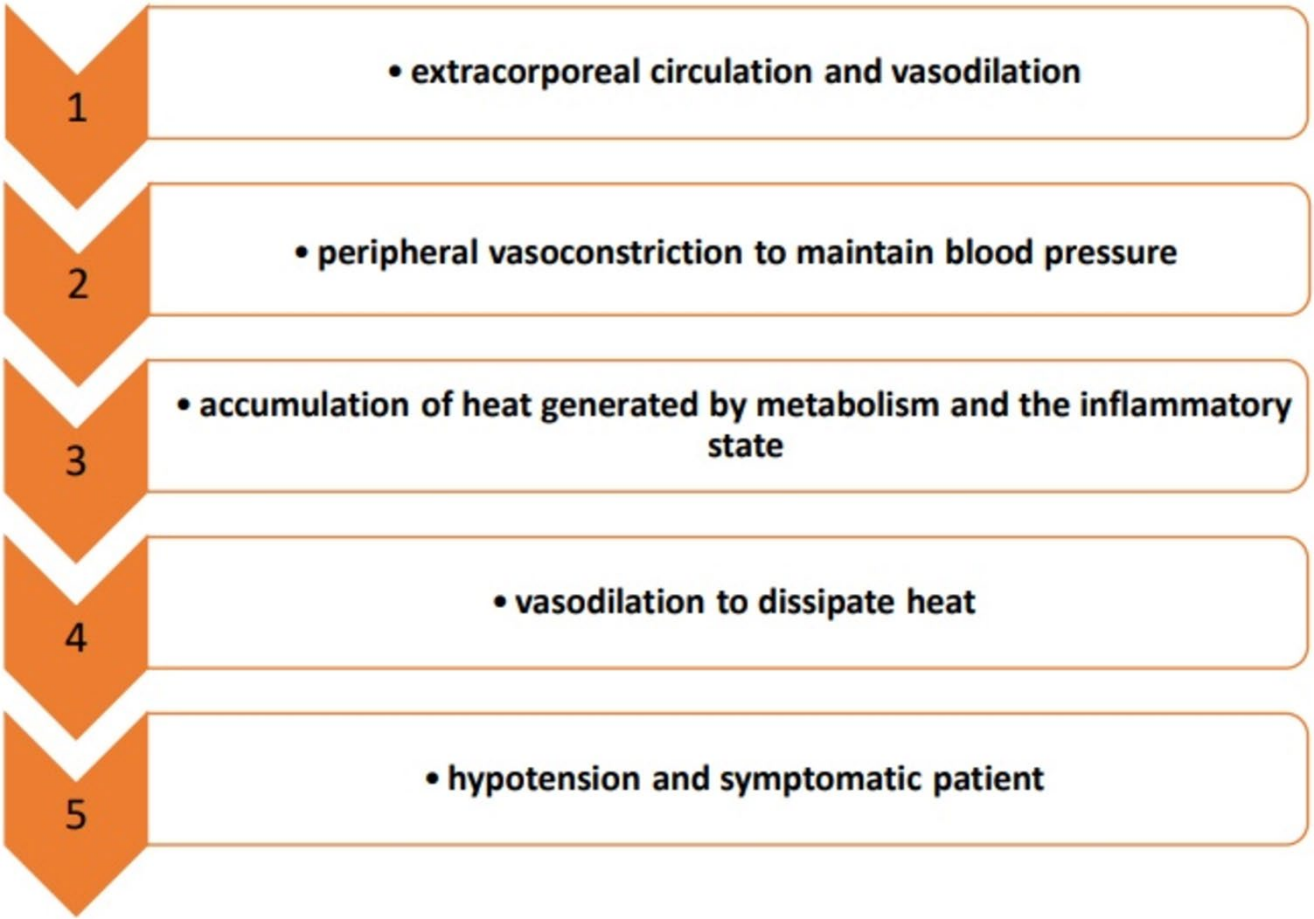
Hemodynamic sequences during hemodialysis. Extracorporeal circulation triggers initial vasodilation, followed by compensatory vasoconstriction. The subsequent accumulation of heat promotes vasodilation to dissipate it, ultimately resulting in hypotension and clinical symptoms

Unclear or suboptimal dialysis can significantly impact the cardiovascular system of patients with end-stage kidney disease (ESKD) through various mechanisms, including episodes of hypotension, myocardial injury, and myocardial stunning (Fig. 1). Additionally, it may affect the central nervous system, leading to neurological complications, and contribute to other clinical effects, such as fatigue and inflammation. Emerging evidence suggests that cool dialysis may play a beneficial role in mitigating some of these adverse effects by improving hemodynamic stability and reducing cardiovascular and neurological risks.

### Myocardial injury and myocardial stunning

**Fig. 2:**
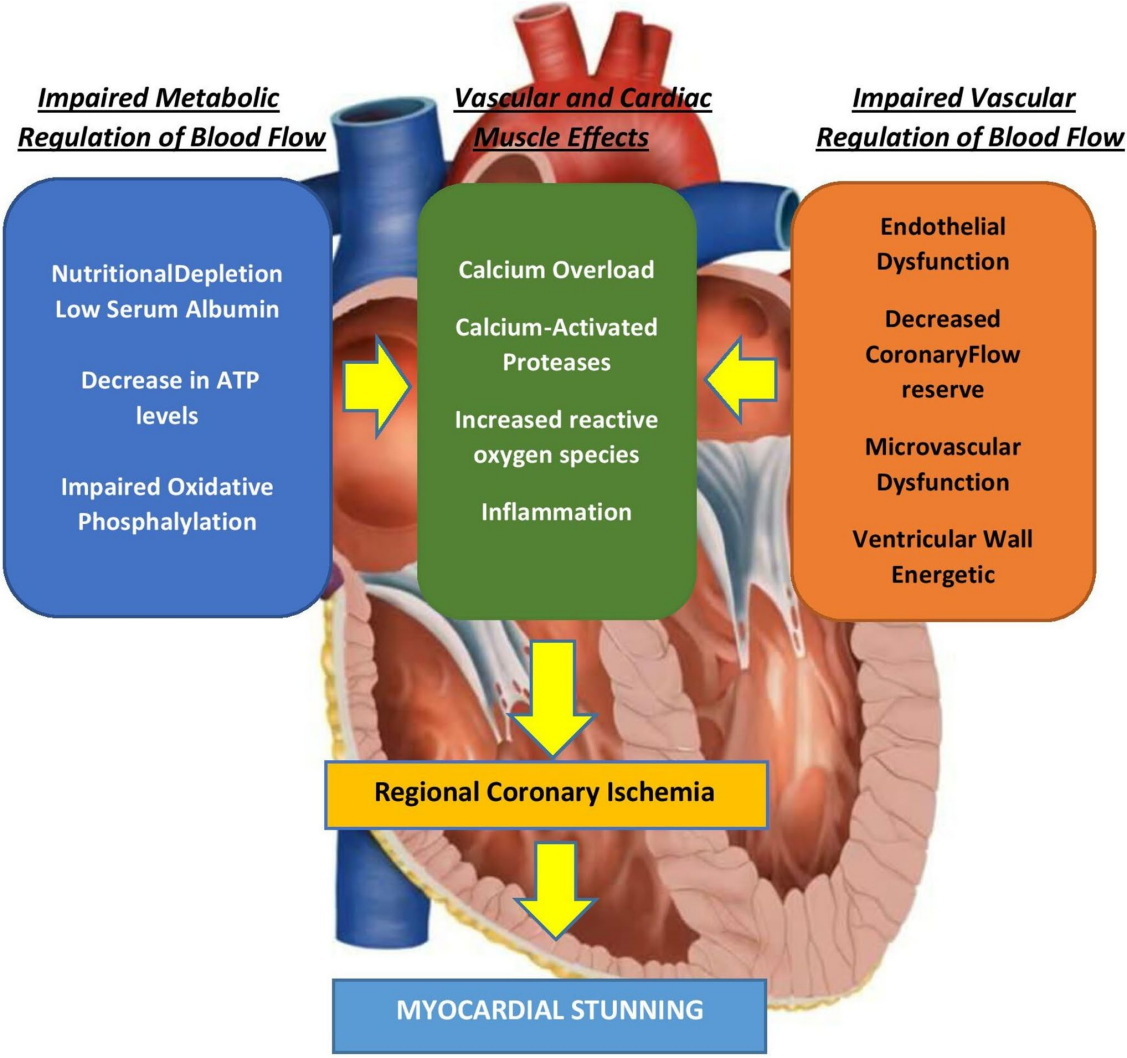
Mechanisms of myocardial stunning in hemodialysis. Impaired metabolism, vascular dysfunction, calcium overload, and oxidative stress converge to induce regional ischemia and transient ventricular dysfunction

In addition to intradialytic hypotension, myocardial damage is strongly influenced by the adaptive responses triggered during hemodialysis. It has been demonstrated that arterial blood pressure may fall by up to 20 mmHg during a standard session, leading to reduced tissue perfusion, particularly at the coronary level, with consequent impairment of myocardial contractility. This phenomenon, known as *myocardial stunning* (Fig. 2), results mainly from a transient reduction in regional blood flow, which can decrease by as much as 30% [6, 7].

Myocardial stunning is the delayed recovery of regional heart muscle contractile function after an ischemia–reperfusion event, even when there is no irreversible structural damage, once baseline coronary flow is restored [8].

Its pathogenesis involves multiple factors, including depletion of high-energy phosphates, microvascular dysfunction, changes in neurosensory responses, calcium homeostasis dysregulation, and elevated reactive oxygen species (ROS) production [9].

### Hemodialysis and tissue hypoperfusion

Hemodialysis-induced hypoperfusion arises from a sequence of events closely linked to extracorporeal circulation (ECC) and the body’s adaptive responses. As illustrated in Fig. 1, the reduction in circulating blood volume initially triggers peripheral vasoconstriction, aimed at maintaining blood pressure and ensuring tissue perfusion. This is later followed by systemic vasodilation, a compensatory mechanism to dissipate the heat generated during treatment. Within this context, thermal modulation of dialysis—known as cool dialysis—has been proposed as a strategy to attenuate vasodilation, improve hemodynamic stability, and reduce the risk of tissue hypoperfusion and organ damage.

Among the hemodynamic changes associated with hemodialysis, intradialytic hypotension (IDH) remains the most detrimental. In patients with coronary artery disease, IDH is a major precipitating factor for myocardial ischemia [10]. However, myocardial stunning may also occur in the absence of pre-existing coronary disease, reflecting a complex and multifactorial pathogenesis.

Increased production of reactive oxygen species (ROS) and chronic inflammation have been identified as major contributors to these alterations, particularly in patients on hemodialysis. In contrast, these mechanisms appear less prominent in peritoneal dialysis [11]. Beyond the cardiovascular system, the central nervous system may also be affected, as hypoperfusion, oxidative stress, and inflammation contribute to structural and functional brain alterations.

### Effects on central nervous system

In addition to myocardial injury, hemodialysis can also exert significant effects on the central nervous system (CNS). Hypoperfusion, oxidative stress, and systemic inflammation contribute to structural and functional alterations, particularly within the cerebral cortex [12].

A study by Yu et al. [13] demonstrated that patients with ESKD on dialysis show a diffuse reduction in cortical thickness and volume compared to healthy controls, with more marked changes in regions such as the paracentral gyrus, transverse temporal gyrus, and occipital cortex. Deep brain structures—including the anterior thalamic radiation, superior longitudinal fasciculus, corticospinal tract, uncinate fasciculus, and nucleus accumbens—are also affected, especially in long-term dialysis patients. Alterations in these regions correlate with measurable cognitive deficits, as assessed by tools such as the Wechsler Intelligence Scale (WAIS) and the Montreal Cognitive Assessment (MoCA) [14, 15]. As with cardiac injury, the etiology appears multifactorial, involving hypoperfusion, systemic inflammation, and circulating endotoxins.

**Fig. 3:**
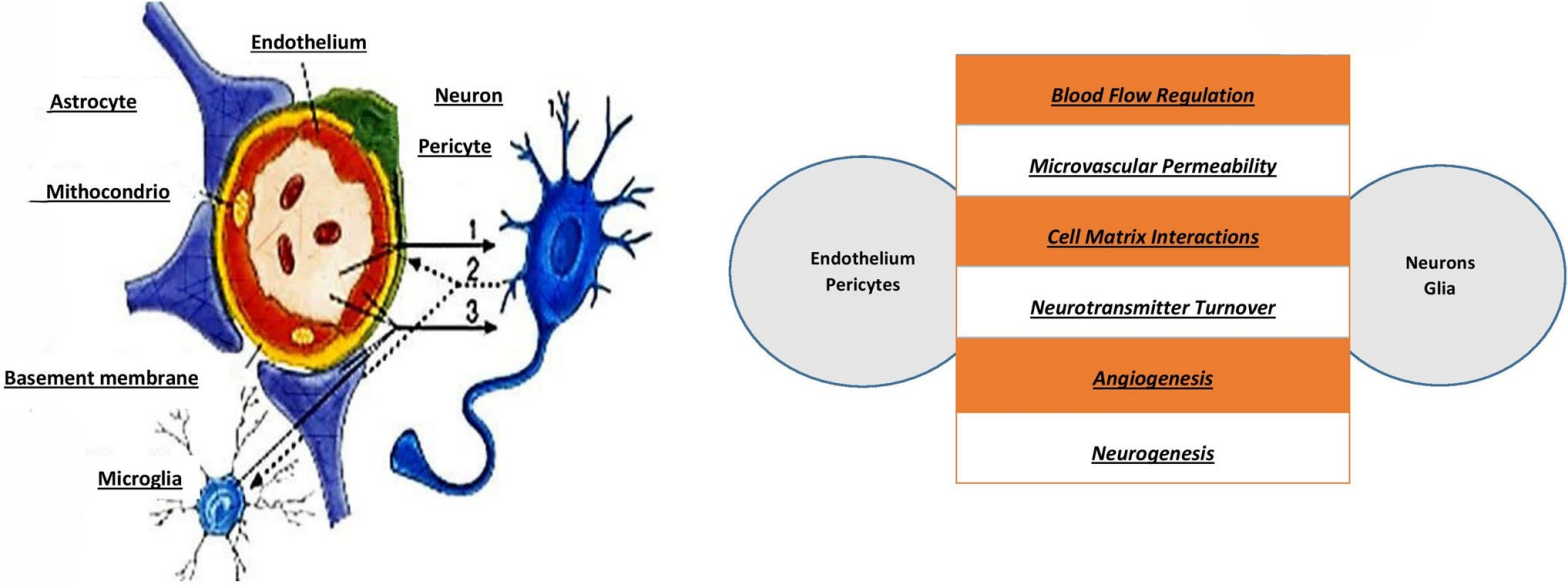
Structure and function of the neurovascular unit. Endothelial cells, pericytes, glial cells, and neurons interact to regulate blood flow, vascular permeability, neurotransmission, and neurogenesis, ensuring cerebral homeostasis

Under physiological conditions, the brain receives approximately 15–20% of cardiac output and maintains stable blood flow across a wide range of systemic pressures through autoregulation by the neurovascular unit, formed by glial cells and vascular structures (Fig. 3). In dialysis patients, however, this ability to regulate vascular tone is impaired, as shown in the Rotterdam Study by Sedaghat et al. [17].

The use of reduced-temperature dialysate has been associated with the attenuation of microvascular injury. As reported by Eldehni et al. [4], lower blood pressure variability and improved hemodynamic stability translate into less ultrastructural brain damage, as demonstrated by neuroimaging. In particular, diffusion tensor magnetic resonance imaging (DTI-MRI) has enabled detailed characterization of microcirculatory alterations in this population [18].

### Other clinical effects

Among the direct consequences of hemodialysis, fatigue during and after the session is one of the most frequently reported symptoms, affecting 60–80% of patients and often persisting for several hours post-treatment [19]. This symptom is multifactorial: rapid fluid and solute shifts, accumulation of pro-inflammatory cytokines, and transient hemodynamic instability all contribute to post-dialysis asthenia, which negatively affects daily functioning and quality of life.

Other clinical manifestations are also linked to the chronic inflammatory state typical of patients on long-term hemodialysis. Persistent inflammation contributes not only to fatigue but also to a greater susceptibility to infections, vascular calcifications, and protein–energy wasting. Furthermore, thermal discomfort and cold sensation, more common during cool dialysis, can occasionally limit patient tolerance and adherence, despite the hemodynamic advantages of this strategy.

Hemodialysis can exacerbate neurovegetative symptoms—such as dizziness, muscle cramps, and headaches—that often occur with intradialytic hypotension, and fluid overload may worsen depression and sleep problems. Dialysis patients commonly face sleep issues like insomnia, restless legs, and daytime sleepiness, caused by fluid shifts, electrolyte imbalances, and coexisting pain or depression. Longer or nocturnal dialysis schedules can disrupt circadian rhythms and reduce light exposure and activity levels, while anemia, pruritus, and medication effects add to the overall burden. Poor sleep then further impairs blood pressure control and glycemic stability, creating a vicious cycle that diminishes the quality of life [20].

These manifestations further underline the need for strategies that improve cardiovascular stability and mitigate treatment-related adverse effects.

### The concept of cool dialysis

The concept of *cool dialysis* was first introduced by Jeffries et al. in 2011 [21] and subsequently developed by Eldehni et al. in 2015 [4]. It refers to a hemodialysis strategy in which the dialysate temperature is set at least 1 °C lower than the standard system set-point of 37 °C. In clinical practice, this corresponds to temperatures between 35 and 36 °C, with the aim of modulating the hemodynamic response and reducing the incidence of intradialytic hypotension.

An alternative approach, proposed by Pizzarelli [22], involves individualizing dialysate temperature based on the patient’s baseline core body temperature. In this protocol, the temperature is set approximately 0.5 °C below the patient’s resting value, as measured before the session. This method seeks to optimize hemodynamic tolerance and minimize blood pressure fluctuations during treatment.

The rationale for this strategy is supported by the observation that standard-temperature dialysis does not ensure isothermia throughout the session. On the contrary, core body temperature often rises by 0.5–0.7 °C during treatment, a condition that can promote recurrent hypotensive episodes and long-term systemic stress.

Before analyzing the clinical effects of low-temperature hemodialysis, it is useful to recall some basic physiological concepts that explain the mechanisms underlying the increase in core body temperature during dialysis.

### Physiology of thermoregulation and pathogenesis of dialysis-induced hyperthermia

Under physiological conditions, core body temperature (*T*_b_) is tightly regulated by the CNS within a narrow range of about 3–4 °C around a mean value of 36.8 °C [23]. In general, body temperature is maintained at 37 ± 0.5 °C (98.6 ± 0.9 °F), an interval considered optimal for metabolic processes and enzymatic function [24]. Homeothermy is ensured by complex regulatory mechanisms located mainly in the hypothalamus, which integrate inputs from peripheral and central thermoreceptors and modulate effector responses to balance heat production and dissipation. Even small deviations from this range can challenge thermoregulation and produce severe clinical consequences: values above 42 °C cause cytotoxicity, protein denaturation, and impaired DNA synthesis, whereas temperatures below 27 °C are incompatible with life [25].

Thermoregulation results from the coordinated activity of specific hypothalamic nuclei, temperature-sensitive receptors, and effector pathways. The supraoptic nucleus functions as a central processor of thermal information. It receives input from peripheral thermoreceptors via the lateral spinothalamic tract. Thermosensory inputs mainly originate from receptors that respond to multiple stimuli, with some specifically tuned to temperature changes. Thermoreceptors are mainly located in the skin and mucosa, but are also found in the walls of visceral organs, muscle tissue, airways, blood vessels, and the spinal cord. These signals are transmitted along peripheral afferents toward the CNS [26, 27].

Based on these inputs, the hypothalamus activates distinct effector pathways in response to high- or low-temperature stimuli, engaging neural circuits that coordinate the central and peripheral responses.

As demonstrated in classical studies by Schnedititz [28] and later expanded by Pizzarelli [29], the rise in body temperature during hemodialysis must be understood as the result of heat transfer across both the extracorporeal circuit and the patient’s circulation, which can be divided into superficial and deep compartments.

**Fig. 4:**
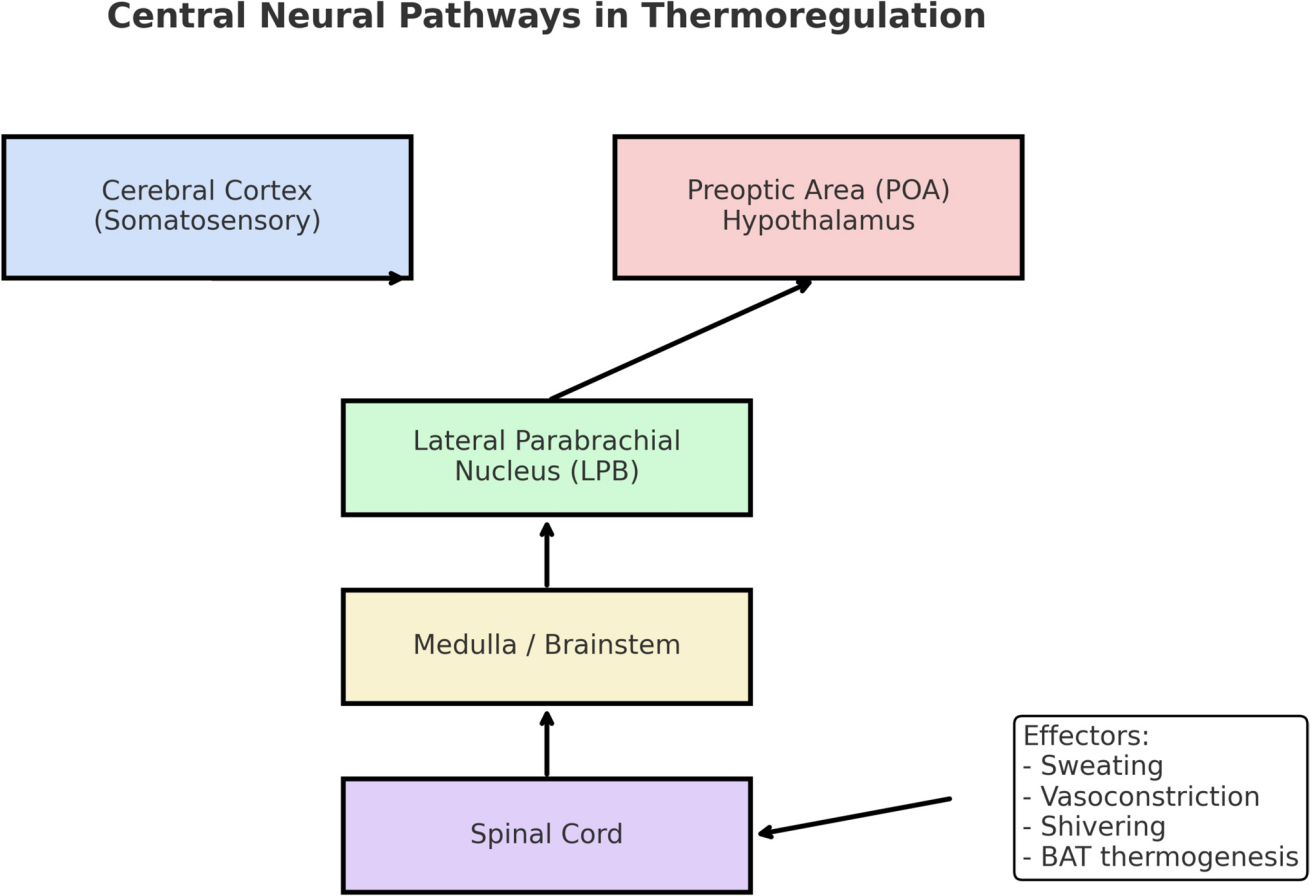
Central neural pathway in thermoregulation. Schematic representation of afferent and efferent circuits integrated by the hypothalamic preoptic area (POA) leading to thermoregulatory responses

Heat transfer during dialysis can be expressed by the following equation (see Fig. 4):

$${\boldsymbol{E}}={\boldsymbol{c}}\times{\boldsymbol{\rho}}\times ({{\boldsymbol{T}}}_{\mathbf{v}\mathbf{e}\mathbf{n}}-{{\boldsymbol{T}}}_{\mathbf{a}\mathbf{r}\mathbf{t}})\times {{\boldsymbol{Q}}}_{\mathbf{b}}$$

in which

***E = ***thermal energy transferred;

***c = ***specific heat constant of blood (amount of heat required to raise the temperature of 1 g of blood by 1 °C);

***ρ = ***blood density per unit volume;

***T***_**ven**_** = **temperature of the blood in the venous line (inflow);

***T***_**art**_** = **temperature of the blood in the arterial line (outflow);

***Q***_**b**_** = **blood pump flow rate.

This formula highlights how heat transfer depends on the temperature gradient between venous and arterial blood, the blood flow rate, and the thermophysical properties of blood. Both Tven and Tart are influenced by multiple factors, including the length and conductivity of extracorporeal lines, ambient temperature, and arteriovenous fistula (AVF) recirculation.

Using dialysate at 37 °C, though justified by its similarity to normal body temperature, oversimplifies the complex thermal dynamics of the extracorporeal system. In fact, temperature varies across different body regions: it is higher than 37 °C in vessels and splanchnic organs, and lower in peripheral tissues. In distal AVFs, for example, there is often a difference of about 2 °C between the radial artery and major veins at the elbow [30].

**Fig. 5:**
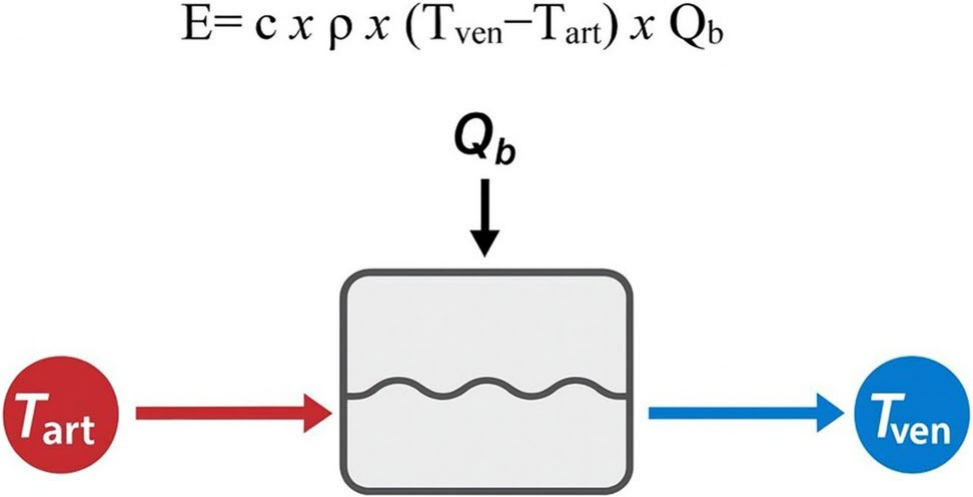
Determinants of heat transfer during hemodialysis. Thermal energy exchange (*E*) is defined by the specific heat capacity of blood (*c*), blood density (*ρ*), temperature gradient between venous (*T*_ven_) and arterial (*T*_art_) blood lines, and blood flow rate (*Q*_b_). This equation highlights the contribution of extracorporeal circulation to the patient’s overall thermal balance during dialysis sessions

Another relevant factor is the relationship among body temperature, dialysate temperature, blood flow rate, and dialysate flow rate. For instance, a patient with a baseline core temperature of 36 °C treated with dialysate at 37 °C, a Qb of 200 mL/min, and a Qd of 450 mL/min will show a progressive rise in body temperature during the session (Fig. 5).

### Evidence from the last decade (2015–2025): clinical trial on cool dialysis

Over the past decade, several clinical studies have explored the impact of low-temperature hemodialysis on hemodynamic stability, cardiovascular outcomes, and neurological function. Overall, the strongest evidence originates from 9 studies (Table 1) that fulfilled the aforementioned inclusion and exclusion criteria. This evidence is heterogeneous: while small-scale randomized trials reliably showed improvements in blood pressure stability and myocardial protection, the outcomes of larger pragmatic studies have been more mixed, emphasizing the challenges in implementing this intervention in routine practice. The first relevant study, conducted by Veerappan et al. [31], investigated the use of individualized low-temperature dialysate (36.5 ± 0.2 °C, range 35.7–37.5) in a prospective crossover design involving 60 patients. The trial demonstrated a clear reduction in intradialytic hypotension (IDH) and attenuated blood pressure variability, suggesting improved hemodynamic tolerance. Although the sample size was modest and the follow-up limited, these findings provided early confirmation of the protective potential of thermal modulation.

The most substantial evidence comes from the trial* “Major Outcomes with Personalized Dialysate Temperature* (MyTEMP)”, a pragmatic cluster-randomized study including over 15,000 patients across 84 dialysis centers in Ontario, Canada, with more than 4.3 million dialysis sessions recorded between 2017 and 2021 [31]. By design, MyTEMP tested the effectiveness of individualized dialysate temperature (0.5–0.9 °C below baseline core body temperature, minimum 35.5 °C) against a standard of 36.5 °C. The primary composite endpoint—cardiovascular death or hospitalization for myocardial infarction, ischemic stroke, or congestive heart failure—showed no significant difference between groups (adjusted HR 1.00; 95% CI: 0.89–1.11; *p* = 0.93). Similarly, blood pressure variability was not significantly reduced. Importantly, patients receiving individualized low-temperature dialysis more frequently reported thermal discomfort, raising concerns regarding tolerability and long-term adherence. These results suggest that while cool dialysis may confer physiological benefits, its widespread adoption in unselected populations remains debatable [32].

To address these limitations, Ouyang et al. [33] conducted a Bayesian reanalysis of the MyTEMP trial, applying Weibull parametric models with different priors to explore the probability of treatment efficacy. This more flexible approach revealed a high probability of clinical benefit, with 96% credible intervals excluding unity, thus supporting the hypothesis that cool dialysis has favorable effects which might have been obscured by the stringent assumptions of frequentist statistics. Taken together, the original MyTEMP and the Bayesian reanalysis highlight the ongoing uncertainty in interpreting large-scale evidence, where statistical methodology can significantly affect clinical conclusions.

In a smaller randomized controlled trial, Odudu et al. [34] assessed personalized dialysate temperature in 73 patients, with endpoints including left ventricular (LV) systolic function and frequency of IDH episodes. Low-temperature dialysis was associated with fewer hypotensive episodes and reduced transient LV dysfunction, consistent with a cardioprotective role. These findings echo earlier insights from the Frequent Hemodialysis Network (FHN) trial [35], which suggested that improved hemodynamic stability contributed to reductions in LV mass and end-systolic volume, despite no change in ejection fraction at 12 months. However, Odudu’s trial lacked the statistical power and clinical endpoints (hospitalization, mortality) needed to generalize these benefits to broader populations.

Regarding neurological outcomes, Dasgupta et al. [36] performed a randomized controlled pilot study comparing cool versus standard dialysate. After 12 months, no significant differences were observed in cognitive performance. Nevertheless, the intervention proved safe and well tolerated, laying the groundwork for larger studies to explore potential neuroprotective effects.

In summary, the available evidence suggests that cool dialysis improves hemodynamic stability and reduces IDH, with consistent cardioprotective signals in small trials. However, large-scale pragmatic evidence remains neutral, and tolerability issues such as thermal discomfort cannot be overlooked. The impact on long-term cardiovascular outcomes and cognitive function is still uncertain, and future research should focus on identifying subgroups of responder patients who are most likely to benefit from this strategy.

## Summary of evidence and conclusions

The evidence reviewed confirms that low-temperature hemodialysis can offer multiple clinical benefits, particularly in terms of hemodynamic stability and prevention of intradialytic hypotension. Small- and medium-sized trials, such as those by Veerappan et al. and Odudu et al., consistently demonstrated fewer hypotensive episodes and a protective effect on left ventricular function. Although limited by study heterogeneity, varying temperature definitions, and follow-up duration, overall [37, 38] these findings support the hypothesis that thermal modulation contributes to improved cardiovascular tolerance during dialysis. The magnitude of the effect of low-temperature dialysis remains uncertain, and the large multicenter MyTEMP trial failed to show significant reductions in major cardiovascular outcomes, highlighting the complexity of translating physiological benefits into hard clinical endpoints. Nevertheless, the Bayesian reanalysis of MyTEMP data suggests a high probability of clinical benefit, underscoring how statistical methodology and study design can influence interpretation.

Similarly, the effects of cool dialysis on the CNS remain uncertain. While neuroimaging studies suggest a potential role in attenuating microvascular damage, clinical trials such as that by Dasgupta et al. have not demonstrated consistent improvements in cognitive outcomes.

Taken together, the data indicate that cool dialysis is not yet justified as a routine strategy for all patients. Instead, its greatest potential appears to lie in selected subgroups—particularly those with recurrent intradialytic hypotension or evidence of cardiovascular vulnerability—who may derive measurable benefit from improved hemodynamic stability.

Given these premises, future research should focus on large, multicenter studies with extended follow-up, capable of assessing the impact of this intervention on major cardiovascular events, hospitalization, mortality, and long-term cognitive decline. Future work should target higher-risk subgroups, such as patients prone to hypotension, and explore innovative blinding strategies [39]. Equally important is the identification of clinical or biomarker-based profiles that may help define the subgroup of responder patients most likely to benefit from this strategy.

Until new evidence becomes available, adopting a personalized and customized approach to dialysis prescription is essential to improve the sustainability of dialysis and, ultimately, patient outcomes.

## Data Availability

No datasets were generated or analyzed during the current study.
